# Application of Lectin Array Technology for Biobetter Characterization: Its Correlation with FcγRIII Binding and ADCC

**DOI:** 10.3390/microarrays6010001

**Published:** 2016-12-24

**Authors:** Markus Roucka, Klaus Zimmermann, Markus Fido, Andreas Nechansky

**Affiliations:** 1 Vela Labs GmbH, Brunner Str. 69/ Obj. 3, 1230 Vienna, Austria; k.zimmermann@vela-labs.at (K.Z.); m.fido@vela-labs.at (M.F.); 2 JHL Biotech, Zhubei City, Hsinchu County 302, Taiwan; anechansky@jhlbiotech.com

**Keywords:** lectin carbohydrates, lectin microarray, glyco-profiling, fucosylation, ADCC, GlycoStation, LecChip, anti-Lewis Y mAb

## Abstract

Lectin microarray technology was applied to compare the glycosylation pattern of the monoclonal antibody MB311 expressed in SP2.0 cells to an antibody-dependent cellular cytotoxic effector function (ADCC)-optimized variant (MB314). MB314 was generated by a plant expression system that uses genetically modified moss protoplasts (*Physcomitrella patens*) to generate a de-fucosylated version of MB311. In contrast to MB311, no or very low interactions of MB314 with lectins *Aspergillus oryzae* l-fucose (AOL), *Pisum sativum* agglutinin (PSA), *Lens culinaris* agglutinin (LCA), and *Aleuria aurantia* lectin (AAL) were observed. These lectins are specific for mono-/biantennary N-glycans containing a core fucose residue. Importantly, this fucose indicative lectin-binding pattern correlated with increased MB314 binding to CD16 (FcγRIII; receptor for the constant region of an antibody)—whose affinity is mediated through core fucosylation—and stronger ADCC. In summary, these results demonstrate that lectin microarrays are useful orthogonal methods during antibody development and for characterization.

## 1. Introduction

Glycosylation is a post-translational modification of most cellular proteins that occurs in the rough endoplasmatic reticulum, cytoplasm, and Golgi apparatus [1] and affects their structure, function, and activity. Diversity in glycosylation exists at every level of biological organization, between species, tissues, cell types, and proteins within the same organism. A typical example for glycosylated proteins are monoclonal antibodies (mAbs) where complex carbohydrate structures are attached to the conserved N-glycosylation site in the Fc-part (constant region of an antibody) of the immunoglobulin G (IgG) molecule [2,3,4]. A large number of therapeutic mAbs are on the market, and biosimilar versions of the first line mAbs have already been approved. Besides other quality attributes, the glycan pattern of individual mAbs and their biosimilars must be adequately analyzed not only to demonstrate comparability to the innovator but also more generally to show batch-to-batch consistency [3,4]. Importantly, carbohydrates like terminal galactose residues, bisecting *N*-Acetylglucosamin (GlcNAc), and core fucose have a critical impact on mAb-mediated effector functions such as ADCC and complement dependent cytotoxicity (CDC) [5].

Regarding the clinical efficacy of therapeutic mAbs, those fully lacking core fucosylation have attracted attention as next-generation approaches because of their improved ADCC [6,7]. As an example, a fucose-free variant of trastuzumab (termed TrasGEX; Glycotope GmbH, Berlin, Germany) binds with greater affinity to crystallizable fragment region Ƴ receptor III (FcγRIII) expressed by human natural killer cells and induces enhanced ADCC against human epidermal growth factor receptor 2 (HER2) over-expressing cells [8]. Furthermore, Genentech (San Francisco, USA) has shown that its glyco-optimized anti-CD20 type II mAb obiniuzumab enhances cell death, ADCC, and overall survival in comparison to rituximab. In 2016, Food and Drug Administration (FDA) has assigned “break-through status” and approved Genentech’s obiniuzumab (Gazyva).

Analysis of antibody glycosylation patterns is thus of utmost importance. Standard techniques are based on high performance liquid chromatography, mass spectrometry, and capillary electrophoresis [3]. Lectins are a class of molecules that specifically bind to the carbohydrate moiety of glycoproteins. In combination with microarrays, lectins have evolved as an additional tool for studying the glycosylation patterns of proteins [9]. 

Lectin microarrays were first reported in 2005 [10] and are prepared by immobilizing various lectins to a solid surface. These plant-derived sugar-binding proteins are generally classified into five groups according to the monosaccharide for which they exhibit the highest affinity: mannose, galactose/*N*-acetylgalactosamine, *N*-acetylglucosamine, fucose, and sialic acid.

Analytes including glycoproteins, whole cells, or bacteria are labeled with a fluorescent dye (or antibody) before loading onto a commercially available lectin microarray [4,10]. Depending on the carbohydrate structures that are attached to the analyte, binding to certain lectins will occur. The latest version of microarrays does not require washing steps; thus, low affinity glycoproteins are detected by the lectin–oligosaccharide interaction. The chip is analyzed via confocal-type fluorescence scanner applying evanescent-field fluorescence technology. This detection method allows sensitive, real-time observation of monovalent lectin–oligosaccharide interactions under equilibrium conditions between association and dissociation (k_on_ and k_off_) rates, which leads to a stable fluorescent signal. 

The lectin array technology has been already applied to study the implication of glycosylation in cancer, bacteria, fungi, stem cell, sperm, and diabetes [11]. The focus of this paper is to demonstrate its suitability to discriminate between a glyco-engineered, de-fucosylated antibody and the parental antibody produced in SP 2.0 cells targeting the same epitope. Additionally, it was investigated whether this technology could support the prediction of certain effector functions such as ADCC. 

## 2. Materials and Methods 

Antibodies were labeled with Cy3 fluorescent dye. Twenty microliters of the sample (with a concentration of 50 µg/mL) were added into a tube containing a 100 μg Cy3 mono-reactive dye pack carrying only one reactive group on each dye molecule for accurate labeling of amine groups (GE Healthcare, Little Chalfont, UK), which was then mixed and centrifuged. The tubes were incubated for 1 h at 25 °C in the dark. Excess free-Cy3 was removed by a desalting spin column (0.5 mL, Thermo Fisher Scientific, Waltham, USA) according to the instructions of the manufacturer. The samples were diluted with a probing solution (GlycoTechnica, Yokohama, Japan) and then applied to a LecChip (GlycoTechnica). Lectin specificities are provided in the supplementary information. Samples on LecChips were incubated at 20 °C for 13 hours, and fluorescence was measured by the GlycoStation Reader 1200 (Glycotechnica). The results were analyzed using GlycoStation ToolsPro Suite 1.5 (Glycotechnica). Additional information about lectin specificities can be found in Table S1.

## 3. Results and Discussion

**Figure 1:**
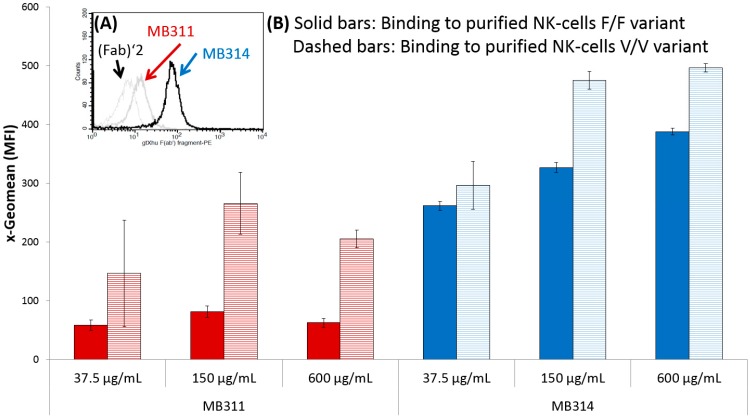
(**A**) Histogram of flow cytometry based binding of MB311 and MB314 to purified Natural Killer (NK) cells. A human (Fab)’2 fragment was used as control. For detection a phycoerythrin (PE)-labeled anti-human F(ab’) fragment was used; (**B**) Three corresponding dilutions (D1–D3) of antibodies (MB311, MB314) were used to show concentration dependent binding to the crystallizable fragment region Ƴ receptor III (FcγRIII) on purified NK cells. Two healthy donors with homogenic phenylalanine/phenylalanine (F/F) (filled bars) and valine/valine (V/V) (shaded bars) variants were used to show the expected difference in binding [14]. MFI: Mean Fluorescent Intensity, MB311■, MB314■.

**Figure 2:**
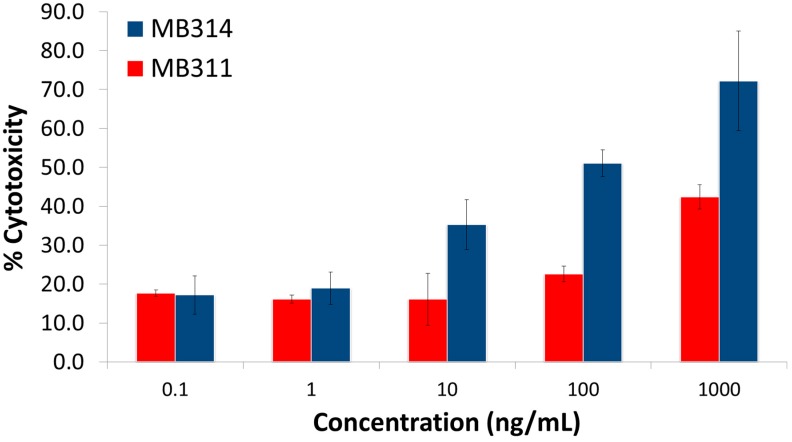
Antibody-dependent cellular cytotoxic effector function (ADCC) activity with purified NK cells from a healthy donor expressing the heterogenic V/F variant of FcγRIII receptor. MB314 induces higher ADCC on SKBR-3 cells than MB311. The error bars show the standard deviation of triplicates. Legend: MB311■, MB314■.

In this study, it was evaluated whether the glycosylation pattern of two mAbs determined by lectin microarray analysis correlated with their effector functions. The pattern of the humanized IgG1/κ anti-Lewis Y mAb MB311 expressed in SP 2.0 cells [12] was compared to the same antibody expressed in a genetically modified, glyco-optimized moss protoplasts expression system (*Physcomitrella patens*). The plant produced de-fucosylated version of MB311 is termed MB314 and showed a concentration dependent binding to FcγRIII (Figure 1). Improved ADCC activity was observed with the dissociation-enhanced lanthanide fluorescence immunoassay (DELFIA)-based system (Perkin Elmer, Waltham, USA) after incubation with europium [13]. The cytotoxicity was calculated based on the controls, where maximum killing was achieved after Triton-X incubation (and set to 100%). Untreated cells were used to assess spontaneous cell death (and set to 0%) (Figure 2) [13]. MB311 has successfully passed clinical Phase I, and MB314 is expected to have superior clinical efficacy [12].

**Figure 3:**
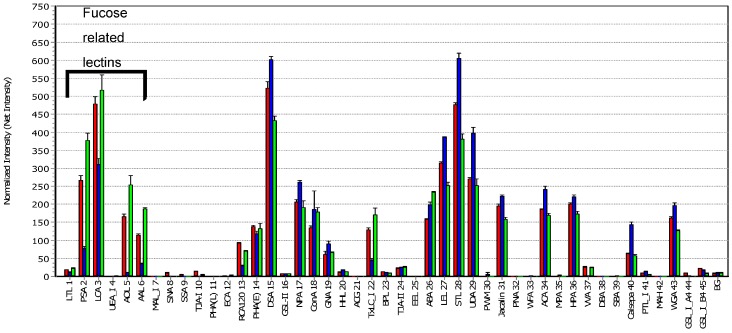
Lectin Array Analysis. Antibodies were labelled by a Cy3 fluorescent dye. All antibodies were diluted, applied to the lectin microarray and measured at different gains. The results were combined with the gain-merging procedure and further normalized by its intensity. To graph the glyco-profile each antibody signal was set in relation to its normalized (averaged) net intensity. The first six lectins are recognizing fucose. The control antibody is a therapeutic antibody of immunoglobulin G1 (IgG1) isotype and was used to show the profile of another fucosylated immunoglobulin. Legend: MB311■, MB314■, and control IgG1■; BG = Background.

For lectin microarray testing, labeled antibodies MB311 and MB314 were applied to a LecChip containing an array of lectins as shown in Figure 3. Fluorescence was measured with the GlycoStation Reader 1200 by applying a gain-merging procedure [15].

The mAbs showed significantly different signals, which can be attributed to the respective glycosylation pattern. The weak signal obtained for RCA120 indicates that MB314 is less galactosylated than MB311. Furthermore, both mAbs are non-sialylated (*Maackia amurensis* agglutinin (MAL-I), *Sambucus nigra* (SNA), *Sambucus sieboldiana* (SSA), *Trichosanthes japonica* agglutinin (TJA-I), and *Agrocybe cylindracea* (ACG)), and MB314 shows a higher content of mannose moieties (*Narcissus pseudonarcissus* (NPA), Concanavalin A (ConA), *Galanthus nivalis* agglutinin (GNA), *Pisum sativum* agglutinin PSA, and *Urtica dioica* agglutinin (UDA)). The differences observed by ConA and Calsepa can also be attributed to different mannose structures. NPA, ConA, GNA, PSA, and UDA represent mannose-binding lectins, but their targets are not necessarily mannosyl glycans. In particular, ConA can bind biantennary complex-type *N*-glycans, even if they have no non-reducing terminal mannose.

The most striking differences were observed with *Aspergillus oryzae* l-fucose (AOL) and with PSA, *Lens culinaris* agglutinin (LCA), and *Aleuria aurantia* lectin (AAL). These lectins have affinity for mono-/biantennary *N*-glycans containing core fucose. In contrast to MB311, no or very low signals were obtained for MB314, thus confirming the expected de-fucosylation. This coincides with the findings of Tateno et al. [16] who reported that LCA and PSA bind specifically to core fucose, whereas AOL and AAL exhibit a broader specificity towards fucosylated glycans. PSA and LCA bind strongest to core-fucosylated biantennary and triantennary *N*-glycans. Thus, their targets are never the high-mannose *N*-glycans which are contained on non-reducing terminus mannose. The lectin binding pattern correlated with MB314 induced higher ADCC against SKBR-3 cells in contrast to MB311 using Natural Killer (NK) effector cells as previously shown (Figure 2) [13]. Moreover, the MB311 and MB314 lectin array data are in alignment with the glycan profile determined previously by Matrix Assisted Laser Desorption/Ionization – Time of Flight Mass Spectrometry (MALDI-TOF) [17], where MB311 was almost completely fucosylated and contained a substantial degree of terminal galactosylation; in MB314, no galactose- or fucose-containing glycan structure albeit minor amounts of IGN314 *N*-glycans terminated in mannose could be detected. Thus, the signals of the above lectins are indicative for core-fucosylated *N*-glycans rather than high-mannose *N*-glycans.

A possible fragment antigen-binding (Fab) glycosylation and potential correlation with cytotoxicity assays, however, was beyond the scope of this study. Usually, glycans of the Fab fragment are described as biantennary complex-type structures that are, in contrast to Fc glycans, highly sialylated. Additionally, high-mannose-type structures can be found on the Fab. Zhang et al. [4] tested the Fab and Fc purified from rituximab and cetuximab, respectively, and confirmed the proper locations of glycosylation sites in Fc or Fab portions. 

In summary, the lectin microarray proved to be a rapid tool for profiling the mAb carbohydrate structures against a broad spectrum of lectins resulting in a glycosylation fingerprint. The analytical sensitivity and sample throughput of lectin microarrays is relatively high; only a very small amount of sample is needed for analysis [11,18]. Thus, this technique has advantages especially in monitoring the glycosylation pattern during process development for recombinant proteins, which depend on various parameters such as medium feeds, metal ions, and harvest time. Results are semi-quantitative, and, for accurate and specific carbohydrate identification, standard methods such as high performance liquid chromatography, mass spectrometry, and capillary electrophoresis should still be applied for confirmation.

Additionally, to support predicting certain effector functions during product development, the positive correlation with increased ADCC and FcγRIII binding of MB314 due to de-fucosylation demonstrates that lectin microarray binding data are a useful surrogate to predict biological functions. 

## Supplementary Materials

The following are available online at http://www.mdpi.com/2076-3905/6/1/1/s1. Additional information about lectin specificities.
