# Supplementary material for: Application of Lectin Array Technology for Biobetter Characterization: Its Correlation with FcγRIII Binding and ADCC
Source: Microarrays (Basel). 2016 Dec 24;6(1):1. doi: 10.3390/microarrays6010001 (PMC5374361; doi:10.3390/microarrays6010001)
Supplement: Supplementary file 1 [file microarrays-06-00001-s001.pdf]

Supplementary Materials: Application of Lectin Array Technology for Biobetter Characterization: Its Correlation with FcγRIII Binding and ADCC

Markus Roucka, Klaus Zimmermann, Markus Fido and Andreas Nechansky

Table S1. Lectin recognition list.

No.	Lectin	Group	Glycan Structure (Major / Strong)	Glycan Structure (Minor / Weak)
Category: Fucose				
1	LTL	Fuc	Fuc a1-2 [H Antigen TypeII, Lewis y],<br>Fuc a1-3 [Lewis x, sialyl Lewis x, Lewis y]	
2	PSA	Fuc<br>N-type	mono/bianntenary N-type with Core Fuc [Fuc a1-6]	Man (2 to high Man)
3	LCA	Fuc<br>N-type	mono/bianntenary N-type with Core Fuc [Fuc α1-6]	Man (6 to high Man), mono/bianntenary agalactosylated N-type without Core Fuc
4	UEA I	Fuc	Fuc a1-2 [H Antigen TypeII]	Fuc a1-4 (Lewis y, Lewis a)
5	AOL	Fuc	Fuc a1-6 [Core Fuc in mono/bi/tri/tetraanntenary N-type], Fuc a1-2 [H antigen TypeII]<br>[1]	Fuc a1-3 [Lewis x, sialyl Lewis x, Lewis y], Fuc a1-4 [Lewis a], Fuc a1-2 [B antigen]
6	AAL	Fuc	Fuc a1-3 [sialyl Lewis x, Lewis x, Lewis y],<br>Fuc a1-2 [H Antigen TypeII]	Fuc a1-6 [Core Fuc in mono/bi/tri/tetraanntenary N-type]
Category: N-Acetylneuraminic acid				
7	MAL I<br>(no data in DB)	NeuAc	NeuAc a2-3Gal<br>[2,3]	
8	SNA	NeuAc	NeuAc a2-6Gal, NeuAc a2-6GalNAc<br>[4]	
9	SSA	NeuAc	NeuAc a2-6Gal	
10	TJA-I	NeuAc	NeuAc a2-6Gal,<br>(HSO3-)-6Gal b1-4GlcNAc<br>[5]	galactosylated N-type
Category: Others(Gal, N-type bisecting)				
11	PHA(L)	N-type	GlcNAc b1-6Man [mainly tetraanntenary N-type]	GlcNAc b1-6Man
12	ECA	Gal<br>N-type	Galactosylated N-type,<br>Gal b1-4GlcNAc	

13	RCA120	Gal<br>N-type	Galactosylated N-type,<br>Gal b1-4GlcNAc	
14	PHA(E)	Others<br>N-type	Bisecting GlcNAc (GlcNAc b1-4Man) in galactosylated N-type	galactosylated N-type without bisecting GlcNAc
15	DSA	N-type	Galactosylated tetraantenary N-type	Galactosylated trianntenary N-type,<br>polylactosamine [(Gal b1-4GlcNAc)n, n>=2]
Category: N-Acetylglucosamine				
16	GSL II	GlcNAc<br>N-type	Agalactosylated tetra/trianntenary N-type<br>[6]	Agalactoslylated bianntenary N-type
Category: Mannose				
17	NPA	Man<br>N-type	monoanntenary N-type (galacto & agalacto),<br>Core Man	Bi/trianntenary N-type(galacto & agalacto),<br>High Man
18	ConA<br>(no data in DB)	Man, Glc	High Man with tetramannnosyl Core [Man a1<br>2Man a1-6(Man a1-3)Man b-]	Core Man [Man a1-6(Man a1-3)Man] [7]<br>mono/bianntenary agalactosylated N-type [8]
19	GNA	Man<br>N-type	Core Man [Man a1-6(Man a1-3)Man],<br>Man (3 to 6), monoanntenary N-type with Core<br>Man (galacto & agalacto)	bi/trianntenary N-type
20	HHL	Man<br>N-type	Core Man [Man a1-6(Man a1-3)Man],<br>monoanntenary N-type	Man (~ High Man),<br>bi/tri/tetraanntenary N-type
Category: Others(Gal, N-type bisecting)				
21	ACG	Gal<br>N-type,<br>Glycolipid	(Gal b1-3Gal)n,<br>NeuAc a2-3Gal (sialylated N-type),<br>*NueAc a2-3Gal b1-3GalNAc b1-4(NeuAc a2-8NeuAc a2-3)Gal b1-4Glc [GT1b],<br>*NueAc a2-3Gal b1-3GalNAc b1-4(NeuAc a2-3)Gal b1-4Glc [GD1a]	Gal b1-4GlcNAc (galactosylated bi/tri/tetraanntenary N-type), Gal a1-3Gal
Category: Fucose				
22	TxLC I	N-type	bi/trianntenary N-type with Core Fuc (galacto &<br>agaracto)	bi/trianntenary N-type without Core Fuc (galacto & agalacto),<br>monoanntenary N-type with Core Fuc
Category: Others(Gal, N-type bisecting)				
23	BPL	Gal	Gal b1-4GlcNAc (galactosylated tri/tetraantenary<br>N-type), Gal b1-4(Fuc a1-3)GlcNAc [Lewis x],<br>*Gal b1-3GalNAc b1-4Gal b1-4Glc [GA1],<br>T antigen	GalNAc a1-3(Fuc a1-2)Gal [A antigen],<br>Gal b1-4GlcNAc (galactosylated bianntenary N-type),<br>Gal a1-3Gal,<br>Tn antigen [9]
Category: Fucose				
24	TJA-II	Others	Fuc a1-2Gal [H Antigen, Lewis y]	GalNAc b1-3Gal
25	EEL<br>(no data in DB)	Gal	Gal a1-3(Fuc a1-2)Gal b1-4GlcNAc [B antigen]	

			Fuc a1-2Gal [H antigen Type Ⅰ, Ⅱ] [10]	
Category: O-type (T, Tn, sialyl-T,disialyl-T)				
26	ABA	Gal, GlcNAc, O-type	Gal b1-3GalNAc a- [T antigen] [11]	mono/bi/trianntenary agalactosylated N-type, disialyl T [9]
Category: N-Acetylglucosamine				
27	LEL	GlcNAc	Polylactosamine [(Gal b1-4GlcNAc)n, n>=3]	GlcNAc b1-4GlcNAc
28	STL	GlcNAc	GlcNAc b1-4GlcNAc	Gal b1-4GlcNAc, A antigen
29	UDA	GlcNAc, Man N-type	GlcNAc b1-4GlcNAc, Man (3 to High)	Gal b1-4GlcNAc
30	PWM	GlcNAc	GlcNAc b1-4GlcNAc	Gal b1-4GlcNAc, Man (N-type)
Category: O-type (T, Tn, sialyl-T,disialyl-T)				
31	Jacalin	Gal O-type	GlcNAc b1-3GalNAc a- [Core3], NeuAc a2-3Gal b1-3GalNAc a- [sialyl T] [12,13]	Gal b1-3GalNAc a- [T antigen (Core1)], GalNAc a- [Tn antigen]
32	PNA	Gal O-type, Glycolipid	Gal b1-3GalNAc a- [T antigen], *Gal b1-3GalNAc b1-4Gal b1-4Glc [GA1], Tn antigen, disialyl T [14,15]	*Gal b1-3GalNAc b1-4(NeuNAc a2-3)Gal b1-4Glc [GM1], *Gal b1-4Glc [GA3]
33	WFA	GalNAc, Gal O-type, Glycolipid	GalNAc b1-4GlcNAc, Gal b1-3(-6)GalNAc *GalNAc b1-4Gal b1-4Glc [GA2] , *GalNAc b1-4(NeuAc a2-3)Gal b1-4Glc [GM2]	*GalNAc a1-3GalNAc b1-3Gal a1-4Gal b1-4Glc [Forssman antigen], *GalNAc b1-3Gal a1-4Gal b1-4Glc [Gb4]
34	ACA (no data in DB)	Gal, GalNAc O-type	Gal b1-3GalNAc a- [T antigen], NeuAc a2-3Gal b1-3 GalNAc a- [sialyl T] [16]	
35	MPA (no data in DB)	Gal, GalNAc O-Type	GalNAc a- [Tn antigen] [17]	Gal b1-3GalNAc a- [T antigen] [18]
36	HPA	GalNAc O-type, Glycolipid	*GalNAc a1-3GalNAc b1-3Gal a1-4Gal b1-4Glc [Forssman antigen]	GalNAc a1-3(Fuc a1-2)Gal [A antigen]
37	VVA	GalNAc O-type, Glycolipid	GalNAc a- [Tn antigen], *GalNAc b1-4Gal b1-4Glc [GA2] [9,19]	*GalNAc b1-4(NeuAc a2-3)Gal b1-4Glc [GM2], *GalNAc b1-3Gal a1-4Gal b1-4Glc [Gb4], *GalNAc a1-3GalNAc b1-3Gal a1-4Gal b1-4Glc [Forssman antigen]
38	DBA	GalNAc O-type, Glycolipid	*GalNAc a1-3GalNAc b1-3Gal a1-4Gal b1-4Glc [Forssman antigen]	*GalNAc b1-4(NeuAc a2-3)Gal b1-4Glc [GM2]

39	SBA	GalNAc<br>O-type,<br>Glycolipid	*GalNAc a1-3GalNAc b1-3Gal a1-4Gal b1-4Glc<br>[Forssman antigen], Tn antigen, GalNAc b1<br>4Gal<br>[9]	Gal a1-4Gal, GalNAc b1-4Gal,<br>GalNAc b1-3Gal, T antigen
Category: Mannose				
40	Calsepa	Man,Glc<br>N-type	Galactosylated bianntenary N-type with bisecting<br>GlcNAc	mono/bianntenary N-type, Man (3 to 8)
Category: O-type (T, Tn, sialyl-T,disialyl-T)				
41	PTL I	GalNAc<br>O-type	Gal a1-3(Fuc a1-2)Gal [B antigen], GalNAc a1-3(Fuc a1-2)Gal [A antigen]	*GalNAc a1-3GalNAc b1-3Gal a1-4Gal b1-4Glc [Forssman antigen],<br>Gal a1-3Gal
42	MAH<br>(MAL II)<br>(no data in DB)	Others<br>O-type	disialyl-T [NeuAc a2-3Gal b1-3(NueAc a2<br>6)GalNAc]<br>[20]	
Category: N-Acetylglucosamine				
43	WGA	GlcNAc	GlcNAc b1-4GlcNAc	Gal b1-4GlcNAc (N-type),<br>Man & GlcNAc complex N-type
Category: O-type (T, Tn, sialyl-T,disialyl-T)				
44	GSL I A4	GalNAc,<br>Gal<br>O-type	GalNAc a- [Tn antigen],<br>*GalNAc a1-3GalNAc b1-3Gal a1-4Gal b1-4Glc<br>[Forssman antigen]<br>[21]	Gal a1-3(Fuc a1-2)Gal [B antigen],<br>sialyl Tn
45	GSL I B4	Gal,<br>GalNAc<br>O-type	Gal a1-3(Fuc a1-2)Gal [B antigen]	Gal a1-3Gal b1-4GlcNAc,<br>*Gal a1-4Gal b1-4Glc [Gb3],<br>Core Man N-type with Core Fuc

The categories in the table above shows large classification, which helps to make the lectin microarray more understandable. The glycan structures marked with an asterisk (*): are specific to glycolipids. In a conventional lectin microarray analysis (i.e., Cy3 labeling onto glycoproteins), refer to this information as a reference of glycan binding specificity of each lectin. The list is supported by references [1-21] and by referring to the lectin frontier database: LfDB [22]. It's not covering all of glycan binding specificities. without omission. Abbreviations: LTL (Lotus tetragonolobus); PSA (Pisum sativum); LCA (Lens culinaris); UEA-I (Ulex europaeus); AOL (Aspergillus oryzae); AAL (Aleuria aurantia; MAL_I (Maackia amurensis); SNA (Sambucus nigra); SSA (Sambucus sieboldiana); TJA-I (Trichosanthes japonica); PHAL (Phaseolus vulgaris); ECA (Erythrina cristagalli); RCA120 (Ricinus communis); PHAE (Phaseolus vulgaris); DSA (Datura stramonium); GSL-II (Griffonia simplicifolia); NPA (Narcissus pseudonarcissus); ConA (Canavalia ensiformis); GNA (Galanthus nivalis); HHL (Hippeastrum hybrid); ACG (mushroom, Agrocybe cylindracea); TxLCI (Tulipa gesneriana); BPL (Bauhinia purpurea); TJA-II (Tanthes japonica); EEL (Euonymus europaeus); ABA (fungus, Agaricus bisporus); LEL tomato, Lycopersicon esculentum); STL (potato, Solanum tuberosum); UDA (Urtica dioica); PWM (pokeweed, Phytolacca Americana); Jacalin (Artocarpus integrifolia); PNA (peanut, Arachis hypogaea); WFA (Wisteria floribunda); ACA (Amaranthus caudatus); MPA (Maclura pomifera); HPA (snail, Helix pomatia); VVA (Vicia villosa); DBA (Dolichos biflorus); SBA (soybean, Dolichos biflorus); Calsepa (Calystegia sepium); PTL-I (Psophocarpus tetragonolobus); MAH (Maackia amurensis); WGA (wheat germ, Triticum aestivum); GSL-I A4 (Griffonia simplicifolia); GSL-I B4 (Griffonia simplicifolia)

References

1. Matsumura, K.; Higashida, K.; Ishida, H.; Hata, Y.; Yamamoto, K.; Shigeta, M.: Mizuno-Horikawa, Y.; Wang, X.; Miyoshi, E.; Gu, J.; Taniguchi, N. Carbohydrate binding specificity of a fucose-specific lectin from Aspergillus oryzae: a novel probe for core fucose. *J. Biol. Chem.* **2007**, *282*,15700–15708.
2. Wang, W.C; Cummings, R.D. The immobilized leukoagglutinin from the seeds of Maackia amurensis binds with high affinity to complex-type Asn-linked oligosaccharides containing terminal sialic acid-linked alpha-2,3 to penultimate galactose residues. *J. Biol. Chem.* **1988**, *263*, 4576–4585.
3. Knibbs, R.N.; Goldstein, I.J.; Ratcliffe, R.M.; Shibuya, N. Characterization of the carbohydrate binding specificity of the leukoagglutinating lectin from Maackia amurensis. Comparison with other sialic acid-specific lectins. *J. Biol. Chem.* **1991**, *266*, 83–88.
4. Shibuya, N.; Goldstein, I.J.; Broekaert, W.F.; Nsimba-Lubaki, M.; Peeters, B.; Peumans, W.J. The elderberry (Sambucus nigra L.) bark lectin recognizes the Neu5Ac(alpha 2–6)Gal/GalNAc sequence. *J. Biol. Chem.* **1987**, *262*, 1596–1601.
5. Yamashita, K.; Umetsu, K.; Suzuki, T.; Ohkura, T. Purification and characterization of a Neu5Ac alpha 2-->6Gal beta 1-->4GlcNAc and HSO3(-)-->6Gal beta 1-->GlcNAc specific lectin in tuberous roots of Trichosanthes japonica. *Biochemistry* **1992**, *31*, 11647–11650.
6. Nakamura-Tsuruta, S.; Kominami, J.; Kamei, M.; Koyama, Y.; Suzuki, T,; Isemura, M.; Hirabayashi, J. Comparative analysis by frontal affinity chromatography of oligosaccharide specificity of GlcNAc-binding lectins, Griffonia simplicifolia lectin-II (GSL-II) and Boletopsis leucomelas lectin (BLL). *J. Biochem.* **2006**, *140*, 285–291.
7. Ohyama, Y.; Kasai, K.; Nomoto, H.; Inoue, Y. Frontal affinity chromatography of ovalbumin glycoasparagines on a concanavalin A-sepharose column. A quantitative study of the binding specificity of the lectin. *J. Biol. Chem.* **1985**, *260*, 6882–6887.
8. Hirabayashi, J.; Kasai, K. Separation technologies for glycomics. *J Chromatogr. B Analyt. Technol. Biomed. Life Sci.* **2002**, *771*, 67–87.
9. Sueyoshi, S.; Tsuji, T.; Osawa, T. Carbohydrate-binding specificities of five lectins that bind to O-Glycosyl-linked carbohydrate chains. Quantitative analysis by frontal-affinity chromatography. *Carbohydr. Res.* **1988**, *178*, 213–224.
10. Petryniak, J.; Goldstein, I.J. Immunochemical studies on the interaction between synthetic glycoconjugates and alpha-L-fucosyl binding lectins. *Biochemistry* **1986**, *25*, 2829–2838.
11. Irazoqui, F.J.; Vides, M.A.; Nores, G.A. Structural requirements of carbohydrates to bind Agaricus bisporus lectin. *Glycobiology* **1999**, *9*, 59–64.
12. Tachibana, K.; Nakamura, S.; Wang, H.; Iwasaki, H.; Tachibana, K.; Maebara, K.; Cheng, L.; Hirabayash,i J.; Narimatsu, H. Elucidation of binding specificity of Jacalin toward O-glycosylated peptides: quantitative analysis by frontal affinity chromatography. *Glycobiology* **2006**, *16*, 46–53.
13. Mahanta, S.K.; Sastry, M.V.; Surolia, A. Topography of the combining region of a Thomsen-Friedenreich-antigen-specific lectin jacalin (Artocarpus integrifolia agglutinin). A thermodynamic and circular-dichroism spectroscopic study. *Biochem. J.* **1990**, *265*, 831–840.
14. Lotan, R.; Skutelsky, E.; Danon, D.; Sharon, N. The purification, composition, and specificity of the anti-T lectin from peanut (Arachis hypogaea). *J. Biol. Chem.* **1975**, *250*, 8518–8523.
15. Swamy, M.J.; Gupta, D.; Mahanta, S.K.; Surolia, A. Further characterization of the saccharide specificity of peanut (Arachis hypogaea) agglutinin. *Carbohydr. Res.* **1991**, *213*, 59–67.
16. Rinderle, S.J.; Goldstein, I.J.; Matta, K.L, Ratcliffe RM.Isolation and characterization of amaranthin, a lectin present in the seeds of Amaranthus caudatus, that recognizes the T- (or cryptic T)-antigen. *J. Biol. Chem.* **1989**, *264*, 16123–16131.
17. Sarkar, M.; Wu, A.M.; Kabat, E.A. Immunochemical studies on the carbohydrate specificity of Maclura pomifera lectin. *Arch. Biochem. Biophys.* **1981**, *209*, 204–218.
18. Wu, A.M. Polyvalent GalNAcalpha1-->Ser/Thr (Tn) and Galbeta1-->3GalNAcalpha1-->Ser/Thr (T alpha) as the most potent recognition factors involved in Maclura pomifera agglutinin-glycan interactions. *J. Biomed. Sci.* **2005**, *12*, 135–152.
19. Puri, K.D. Gopalakrishnan B, Surolia A.Carbohydrate binding specificity of the Tn-antigen binding lectin from Vicia villosa seeds (VVLB4). *FEBS Lett.* **1992**, *312*, 208–212.

20. Konami, Y.; Yamamoto, K.; Osawa, T.; Irimura, T. Strong affinity of Maackia amurensis hemagglutinin (MAH) for sialic acid-containing Ser/Thr-linked carbohydrate chains of N-terminal octapeptides from human glycophorin A. *FEBS Lett.* **1994**, *342*, 334–338.
21. Wu, A.M.; Wu, J.H.; Chen, Y.Y.; Song, S.C.; Kabat, E.A.Further characterization of the combining sites of Bandeiraea (Griffonia) simplicifolia lectin-I, isolectin A(4). *Glycobiology* **1999**, *9*, 1161–1170.
22. Lectin frontier database: LfDB. Available online: http://acgg.asia/lfdb2/ (accessed on 3 October 2016).
